# Is Cholecystectomy in Patients with Symptomatic Uncomplicated Cholelithiasis Beneficial in Improving the Lipid Profile?

**DOI:** 10.7759/cureus.6729

**Published:** 2020-01-21

**Authors:** Adel Osman, Arwa H Ibrahim, Areej M Alzamil, Abdullah M Alkhalifa, Dania A Badghaish, Faisal H Al-dera, Reda A Alwosaibi

**Affiliations:** 1 Surgery, Imam Abdulrahman Bin Faisal University, Dammam, SAU; 2 Internal Medicine, Imam Abdulrahman Bin Faisal University, Dammam, SAU; 3 Surgery, King Fahad University Hospital, Dammam, SAU

**Keywords:** cholecystectomy, gallstone disease, lipid profile

## Abstract

Introduction

Gallstone disease is an emerging health issue worldwide with its incidence on the rise. The development of gallstone disease is multifactorial, with risk factors including increased age, female sex, obesity, and the use of oral contraceptive pills. It has been established that more than 50% of patients with gallstone disease have a coexisting lipid disorder. Cholecystectomy, the definitive management of gallstones, may improve the lipid profiles of some patients.

Objectives

This study aims to examine the postoperative changes in the lipid profiles of patients who underwent cholecystectomy. These lipid profiles include levels of low-density lipoprotein (LDL), triglycerides (TG), high-density lipoprotein (HDL), total cholesterol (TC), and the Chol/HDL ratio.

Methods

This retrospective study included 55 patients who underwent cholecystectomy between 2013 and 2017. Biochemical parameters, which include LDL, TG, HDL, and TC levels, were collected using the hospital’s recording system, in addition to the calculation of the Chol/HDL ratio.

Results

Statistically significant changes included a reduction in the mean LDL values in the two-, four-, and six-month postoperative periods (P = 0.029, 0.000, and 0.008, respectively), increased mean TG levels one-week postoperatively (P = 0.034), decreased mean TC levels at four (P = 0.049) and six months (P = 0.026) after cholecystectomy, and increased Chol/HDL ratio at two and 12 months postoperatively (P = 0.03, and 0.022, respectively).

Conclusions

From the results, it can be concluded that cholelithiasis is associated with abnormal lipid profiles and that undergoing cholecystectomy may improve them and reduce the future risk of developing coronary artery disease. However, further research is needed to confirm this association.

## Introduction

Gallstones disease is a chronic recurrent hepatobiliary disease. It is one of the emerging health problems worldwide and is becoming more common with an incidence of 1.4 per 100 persons [1]. Biliary stones are categorized into three main types: cholesterol, pigment, or mixed. Biliary stones formation is a complex process. It is governed by major factors: supersaturation of bile secreted, concentration of bile in the gallbladder, crystal nucleation, and abnormal gall bladder emptying [1]. Gallstone disease is an interaction between genetic and environmental factors. The prevalence increases with age; as an aging population, this justifies the rise in its rate. Females carry a higher incidence by two to three folds because of sex steroids and pregnancy changes. Oral contraceptives increase cholesterol secretion and decrease bile acids, resulting in the supersaturation of bile and increasing lithogenicity [2]. Presentation differs among patients, from asymptomatic and discovered incidentally. The most prevalent symptom is severe abdominal pain, requiring investigations and treatment. By the time they become symptomatic, many patients need surgical intervention. Based on the evidence, more than 50% of patients with gall stones have some sort of lipid disorder [1]. Though lipid and bile acids metabolisms are functionally correlated, how cholecystectomy affects lipid profile is not well-comprehended. High lipid profile readings, consisting of elevations in chylomicron, low-density lipoprotein (LDL), very-low-density lipoprotein (VLDL), and intermediate-density lipoprotein (IDL) levels, are becoming increasingly prevalent, especially with the spreading factors among the Saudi population, such as urban residence, increasing age, especially 40 years; physical inactivity, overweight and obesity, diabetes mellitus, frequent fast food consumption, and so on [3]. This, in turn, raises concerns about the effective management of such conditions. Cholecystectomy, which is considered the definitive management of symptomatic gallbladder diseases, for example, cholecystitis, biliary colic, gallbladder stones, and so on, was shown to decrease total serum lipids, serum cholesterol, serum LDL cholesterol, and serum triglycerides, and increase serum HDL cholesterol post-cholecystectomy after one day, one week, one month, and one year, according to Ali et al., Jindal et al., and Shen et al. [1,4-6]. Thus, the changes in plasma lipids are likely to have a significant effect on lowering the incidence of coronary artery disease development. Since the relationship between cholecystectomy and serum lipid profile has not been established yet, this study will investigate the serum lipid profiles of patients up to 12 months post-cholecystectomy.

## Materials and methods

This retrospective study was carried out in the general surgery department at King Fahd Hospital of the University (KFHU) in Khobar, Saudi Arabia, from 2013 to 2017. The study was approved by the Institutional Review Board Committee of KFHU.

Fifty-five patients in the age group of 30-50 years, of both sexes, who underwent cholecystectomy were enrolled in this study. Patients with liver disease, nephrotic syndrome, diabetes mellitus, or familial dyslipidemias, those who didn’t complete a follow-up of 12 months, and those who have a missing lipid profile were excluded from this study. Data were collected in regard to the lipid profile (LDL - TG - HDL - TC - Chol/HDL ratio) using the hospital’s recording system. Preoperative (baseline), one week, two months, four months, six months, 8-months, 10 months, and 12 months are the intervals used for the lipid profile analysis.

Data analysis was performed using IBM SPSS Statistics for Windows, version 20 (IBM Corp., Armonk, NY). All categorical variables, including gender, nationality, and age groups, were expressed as frequencies and percentages. Mean and standard deviation were calculated for lipid profiles, including LDL, HDL, total cholesterol, triglycerides, and Chol/HDL. The paired sample student t-test was used to compare the baseline mean values of the lipid profile with different follow-ups. P-value < 0.05 was considered significant.

## Results

Demographic data

As shown in Table 1, of the 55 included patients, there were 17 males (30.9%) and 38 females (69.1%). The mean age of the patients was 46.5 ± 13.3 years. Eight patients (14.5%) aged 30 years and younger, 24 patients (43.6%) aged from 31 to 50 years, and 23 patients (41.8%) were older than 50 years. The nationalities included were Saudi (42 patients, 76.4%), Egyptian (5 patients, 9.1%), Filipino (3 patients, 5.5%), Pakistani (2 patients, 3.6%), and other (3 patients, 5.5%).

**Table 1:** Demographic characteristics of cases (n = 55)

	Frequency	Percent
Gender		
Male	17	30.9
Female	38	69.1
Age (years)		
< 30	8	14.5
31 - 50	24	43.6
> 50	23	41.8
Nationality		
Saudi	42	76.4
Egyptian	5	9.1
Filipino	3	5.5
Pakistani	2	3.6
Others	3	5.5

Biochemistry

Data were analyzed at baseline (preoperative) and the following postoperative intervals: one week, two months, four months, six months, eight months, 10 months, and 12 months, as shown in Table 2 and Table 3.

**Table 2:** Lipid profile before and after surgery (n = 55) LDL: low-density lipoprotein; HDL: high-density lipoprotein

	(Min - Max) Mean ±SD				
	LDL	HDL	Total Cholesterol	Triglycerides	Chol/HDL
Baseline	120 ±38.2 (36 - 193)	45.8 ±11.9 (26 - 77)	180 ±51.6 (34 - 271)	128.1 ±66.5 (41 - 382)	4.33 ±1.42 (2 - 8.3)
After 1 week	115.8 ±36.7 (50 - 179)	42.7 ±11.2 (19 - 67)	176.3 ±45.8 (101 - 256)	139.2 ±62.4 (75 - 328)	4.45 ±1.69 (2.3 - 8.6)
After 2 months	101.2 ±39.4 (45 - 178)	46.2 ±15.1 (28 - 80)	178.5 ±52.8 (98 - 246)	123.6 ±41.7 (23 - 186)	3.92 ±1.22 (2.4 - 6.9)
After 4 months	108.4 ±40.8 (41 - 165)	50 ±14.4 (30 - 69)	170.5 ±45.6 (93 - 219)	93 ±24.2 (65 - 141)	3.53 ±0.98 (2.3 - 5.5)
After 6 months	108.5 ±41.3 (43 - 172)	46.2 ±13.6 (29 - 76)	173.2 ±51.1 (100 - 268)	115.5 ±62.1 (45 - 263)	3.96 ±1.34 (2.3 - 6.5)
After 8 months	104.6 ±32.9 (38 - 158)	50.4 ±10 (36 - 72)	173 ±41.8 (84 - 232)	116.7 ±51.2 (50 - 203)	13.63 ±24.69 (2.3 - 82)
After 10 months	93.7 ±33.1 (53 - 158)	47.5 ±14.3 (28 - 73)	159.9 ±39.7 (100 - 229)	114.4 ±46.1 (55 - 177)	3.53 ±0.89 (2.3 - 4.8)
After 12 months	114.4 ±36.9 (54 - 176)	46 ±11.5 (30 - 68)	176.6 ±45 (112 - 251)	116.1 ±46.2 (55 - 204)	3.92 ±0.96 (2.5 - 5.6)

**Table 3:** Pairwise comparison of lipid profile before and after surgery (n = 55)

	P-Values*				
	LDL	HDL	Total Cholesterol	Triglycerides	Chol/HDL
Baseline Vs 1 week	0.35	0.089	0.343	0.034	0.186
Baseline Vs 2 Months	0.029	0.124	0.716	0.204	0.03
Baseline Vs 4 Months	0.082	1	0.049	0.968	0.826
Baseline Vs 6 Months	0.008	0.54	0.026	0.844	0.191
Baseline Vs 8 Months	0.18	0.429	0.568	0.551	0.241
Baseline Vs 10 Months	0.077	0.563	0.82	0.75	0.104
Baseline Vs 12 Months	0.328	0.849	0.955	0.168	0.022

Serum LDL Cholesterol

Statistical significance was found at the two, four, and six-month intervals (P = 0.029, 0.000, and 0.008, respectively). The baseline serum LDL ranged from 36 - 193 mg/dL, with a mean of 120 ± 38.2 mg/dL. The two months’ range was 45 - 178 mg/dL with a mean of 101.2 ± 39.4 mg/dL. The four months’ range was 41 - 165 mg/dL, with a mean of 108.4 ± 40.8 mg/dL, and the six months range was 43 - 172 mg/dL, with a mean of 108.5 ± 41.3 mg/dL.

Triglycerides

Triglycerides was the first element of the lipid profile to change, and it showed a statistically significant increase one week postoperatively, as its range was 75 - 328 mg/dL, with a mean of 139.2 ± 62.4 mg/dL (P = 0.034) while the baseline ranged from 41 - 382 mg/dL, with a mean of 128.1 ± 66.5 mg/dL.

Serum HDL Cholesterol

Although differences between the pre- and postoperative HDL were not statistically significant (P > 0.05), HDL preoperatively ranged from 26 - 77 mg/dL, with a mean of 45.8 ± 11.9 mg/dL, and deceased slightly one week postoperatively, range 19 - 67 mg/dL, with a mean of 42.7 ± 11.2 mg/dL (P = 0.089).

Total Cholesterol

The TC baseline ranged from 34 - 271 mg/dL, with a mean of 180 ± 51.6 mg/dL. There were statistically significant decreases in the four and six-month periods postoperatively. The four months’ range was 93 - 219 mg/dL, with a mean of 170.5 ± 45.6 (P = 0.049), whereas the six-months’ range was 100 - 268 mg/dL, with a mean of 173.2 ± 51.1 (P = 0.026).

Chol/HDL Ratio

The mean baseline of the Chol/HDL ratio was 4.33 ± 1.42 and ranged from 2 - 8.3. It had a statistically significant increase in the two and 12-month postoperative periods, as its mean value in the two-month interval was 3.92 ± 1.22, and it ranged from 2.4 - 6.9. The mean Chol/HDL ratio at 12 months ranged from 2.5 - 5.6, with a mean of 3.92 ± 0.96 (P = 0.03 and 0.022, respectively).

## Discussion

Gallstone disease is a multifactorial disorder caused by the interaction between environmental and genetic factors. In the present study, the following risk factors have been discussed: gender, age, and lipid profile abnormality.

In this study, females were more affected than males with a 2:1 ratio, which is consistent with other studies and explains the consideration of female gender as a risk factor for gallstone disease development [2,7].

Regarding age, the literature proved that increasing age is considered a risk factor for gallstone disease. This study showed that the majority of patients were aged from 31 to 50 years old. This is consistent with the results of Ahi in which the majority of patients were in the fourth decade, followed by the third and fifth decades [1].

LDL cholesterol levels decreased post-cholecystectomy and were found to be statistically significant at two, four, and six months postoperatively. In agreement with previous results, which found a decrease within three days to six months [1,4-10]. However, in the articles by Haq and Sabanathan et al., there was a significant increase [3,11]. This decrease is explained by the increased secretion rate of bile acid and phospholipid postoperatively and the upregulation of the LDL-Apolipoprotein B (ApoB) receptor resulting in LDL endocytosis from the blood into hepatocytes [12]. Within the hepatocytes, LDL binds to lysosomes that induce cholesterol delivery to the intracellular cholesterol pool for bile synthesis, leading to a steady LDL reduction post-cholecystectomy.

Triglyceride levels were found to be significantly increased at one week postoperatively, with a statistically insignificant decrease at the one-month interval and thereafter. Interestingly, Kuldip Singh Ahi had similar findings of an increase in TG after one week postoperatively, and a significant decrease was observed one month postoperatively [1]. The increase in TG 1-week post-cholecystectomy may have resulted from one of three mechanisms:

A. Increased TG use as a source of energy during the catabolic phase of trauma and limited carbohydrates amounts

B. Increased catecholamine release that occurs due to either surgical trauma or in response to types of general anesthesia, which activate hormone-sensitive triacylglycerol lipase (HSL) leading to lipolysis, thus increasing glycerol and TG [13]

C. Lipolytic rate acceleration in subcutaneous adipose tissue in response to a perioperative glucose infusion that overrides the anti-lipolytic effect of the glucose-induced hyperinsulinemia; this indicates a high association between abdominal surgery and lipolysis [13]

The postoperative changes of HDL were not statistically significant, although other studies found significant increases in HDL post-cholecystectomy [1,7,9].

The decrease in serum total cholesterol was found significant at four and six months following cholecystectomy. Similarly, some articles found a decrease in serum total cholesterol at intervals ranging from three days to one month [1-2,5,7,9-10]. Conversely, Sabanathan et al. found a significant increase in total cholesterol levels postoperatively [11]. This accounted for the fact that TC is the precursor for bile acids, whose secretion rate significantly increased along with phospholipids after cholecystectomy, causing improvement in the bile composition and significantly decreased the bile acids pool that may occur as a result of rapid cycling around the enterohepatic circulation (EHC)/hour [12]. Additionally, the upregulation of Apo B/E receptors that may increase the endocytosis of cholesterol and other types of fat into hepatocytes leads to the further formation of bile acids.

The Chol/HDL ratio was significantly higher at the one-week interval and conversely decreased at the one-year interval. The results of Jindal et al. and Alkataan et al., however, indicated that the Chol/HDL ratio was significantly reduced at the one-week and one-month intervals [5,7].

It is worth noting that some studies found that patients gained weight and had a higher body mass index (BMI) after cholecystectomy. This may have been caused by increased caloric, lipid, and carbohydrate intake, which consequently led to worsening lipid profiles [4,14].

It is a well-known fact that disturbances in the lipid profile, specifically hypercholesterolemia, hypertriglyceridemia, and low HDL levels, are associated with an increased risk of coronary artery disease (CAD) and stroke, raising questions on whether an improvement in lipid profile following cholecystectomy may help in the future prevention of CAD and stroke. This was discussed by Alkataan et al., who calculated the atherogenic index pre- and post-cholecystectomy and found it to decrease significantly [7]. On the other hand, cholecystectomy has been found to be associated with increased cardiovascular risk factors. This could be explained by a strong association with metabolic syndrome [6,15].

## Conclusions

Cholelithiasis is associated with an abnormal lipid profile that significantly improved after cholecystectomy. This improvement might continue with time; thus, it might be helpful in the prevention of subsequent CAD development. Therefore, the patient was advised to undergo cholecystectomy.
